# The characterization of hippocampal theta-driving neurons — a time-delayed mutual information approach

**DOI:** 10.1038/s41598-017-05527-2

**Published:** 2017-07-17

**Authors:** Songting Li, Jiamin Xu, Guifen Chen, Longnian Lin, Douglas Zhou, David Cai

**Affiliations:** 1 Courant Institute of Mathematical Sciences and Center for Neural Science, New York University, New York, NY United States of America; 2 Shanghai Key Laboratory of Brain Functional Genomics (Ministry of Education), School of Life Science and the Collaborative Innovation Center for Brain Science, Institute of Brain Functional Genomics, East China Normal University, Shanghai, China; 3 School of Mathematical Sciences, MOE-LSC, and Institute of Natural Sciences, Shanghai Jiao Tong University, Shanghai, China; 4 NYUAD Institute, New York University Abu Dhabi, Abu Dhabi, United Arab Emirates

## Abstract

Interneurons are important for computation in the brain, in particular, in the information processing involving the generation of theta oscillations in the hippocampus. Yet the functional role of interneurons in the theta generation remains to be elucidated. Here we use time-delayed mutual information to investigate information flow related to a special class of interneurons—theta-driving neurons in the hippocampal CA1 region of the mouse—to characterize the interactions between theta-driving neurons and theta oscillations. For freely behaving mice, our results show that information flows from the activity of theta-driving neurons to the theta wave, and the firing activity of theta-driving neurons shares a substantial amount of information with the theta wave regardless of behavioral states. Via realistic simulations of a CA1 pyramidal neuron, we further demonstrate that theta-driving neurons possess the characteristics of the cholecystokinin-expressing basket cells (CCK-BC). Our results suggest that it is important to take into account the role of CCK-BC in the generation and information processing of theta oscillations.

## Introduction

In the rodent hippocampus, the theta wave (4–12 Hz) is a dominant rhythm of local field potential (LFP) during ongoing locomotive activities such as active exploration (AE)^1–3^ and rapid-eye-movement (REM) sleep^4–6^. Theta waves have been associated with various cognitive functions, in particular with learning^7, 8^, memory^9–11^, and spatial navigation^12, 13^. Despite the functional importance of theta oscillations^14–17^, the mechanism underlying the theta generation remains to be elucidated. The medial septum-diagonal band of Broca has been commonly accepted as the ultimate generator of theta waves^18^. However, how theta waves emerge from neuronal activities is yet to be clarified.

Interneurons in the hippocampus may play an important role in the generation of theta waves^19–21^. In early studies, according to characteristic firing patterns relative to theta waves, inhibitory theta neurons were first identified in the hippocampal CA1 area of freely behaving rats^22–24^. Later many other types of interneurons were identified whose temporal firing activities correlate with theta waves in both anesthetized and freely behaving animals^25–27^. In particular, various types of interneurons, including basket cells (BC), axo-axonic cells (AAC), bistratified cells (BIC), oriensalveus/lacunosummoleculare cells (OLM), and cholecystokinin-expressing basket cells (CCK-BC), have been observed to fire phase-locked to theta waves with different phases^27^, suggesting their distinct dynamical roles in information processing.

In a recent experiment, a special class of interneurons, referred to as the theta-driving neuron, has been recorded in the hippocampal CA1 area of freely behaving mice^28^. These neurons fire in a rather reliable theta-bursting pattern with high firing rates around 90 Hz during active exploration and they fire preferentially locked to the ascending phase of theta waves. In contrast to other theta-locked interneurons, only theta-driving neurons show strong Granger causal (GC) influence on the theta band of LFP, and this influence persists over various behavioral states. In general, GC analysis is limited to linear systems^29, 30^; it is yet to assess the validity of its application to neuronal systems as they can be highly nonlinear^31^. However, the GC result suggests the possibility that theta-driving neurons are involved in the generation of theta oscillations. To further ascertain this possibility, it is necessary to devise a novel data analysis to circumvent the limitation of the GC analysis.

In this work, we apply time-delayed mutual information to investigation of the relation between theta-driving neurons and theta waves. In contrast to GC, as it can only capture linear features of a system, this information theoretic approach is valid in any systems even when the underlying interactions exhibit substantial nonlinearity. According to our data analysis results at present, we find that, insensitive to behavioral states, theta waves only share high mutual information with the firing activity of theta-driving neurons. In contrast, there is little mutual information shared between theta waves and non-theta-driving interneurons, and between theta-driving neurons and other bands of LFP oscillations. The direction of information flow suggests an information transfer from the firing activity of these theta-driving neurons to the theta wave, contributing predominantly to the generation of theta oscillations.

As mentioned above, various types of interneurons, including BCs, BICs, AACs, OLMs, and CCK-BCs, fire phase locked to theta waves, sharing similar firing features with theta-driving neurons recorded in our experiment^28^. Unlike theta-driving neurons, these types of interneurons have been well characterized based on their bio-markers, the location of their cell bodies, and the target location of their axons. In our previous experiment^28^, theta-driving neurons have been identified based on their *in vivo* firing activity only. The question of which type of interneurons theta-driving neurons belong to is yet to be examined. Here we perform realistic neuron simulations to investigate the identity of theta-driving neurons from a candidate set of five interneuron types of BCs, BICs, AACs, OLMs, and CCK-BCs. Our simulation results are consistent with the scenario that theta-driving neurons are CCK-BCs involving in the generation and information processing of theta oscillations.

## Results

In this section, we first investigate the relationship between theta-driving neurons and theta waves. We then address the question of which interneuron type theta-driving neurons belong to via realistic simulations of a CA1 pyramidal neuron model. We compute the time-delayed mutual information for causal inference between the experimentally measured theta wave and firing activity of a theta-driving neuron, as well as those between the experimentally obtained theta wave and the simulated subthreshold membrane potential of the realistic pyramidal neuron model induced by a theta-driving neuron.

### Interaction between theta-driving neurons and theta waves

Among the ~150 theta-locked putative interneurons recorded from the mouse hippocampal CA1 area, six theta-driving neurons were identified during mouse exploration^28^. A GC analysis^29^ was employed to distinguish theta-driving neurons from other theta-locked interneurons^28^. It was shown^28^ that theta-driving neurons could generate strong Granger causal influence from their firing activity to LFP signal within the theta band. In contrast, the firing activity of other theta-locked interneurons were influenced by LFP signal in the GC sense. Note that GC analysis is in general applicable to linear systems^30^ while the interaction between neuronal activity and LFP signal can be highly nonlinear^31^. Therefore, further investigation of the validity of the interpretation of the GC results is required. For instance, how strongly do theta-driving neurons interact nonlinearly with theta waves? How to characterize consequences of this nonlinear interaction? In which way do theta-driving neurons *drive* the theta wave? We attempt to address these questions in this section.

**Figure 1:**
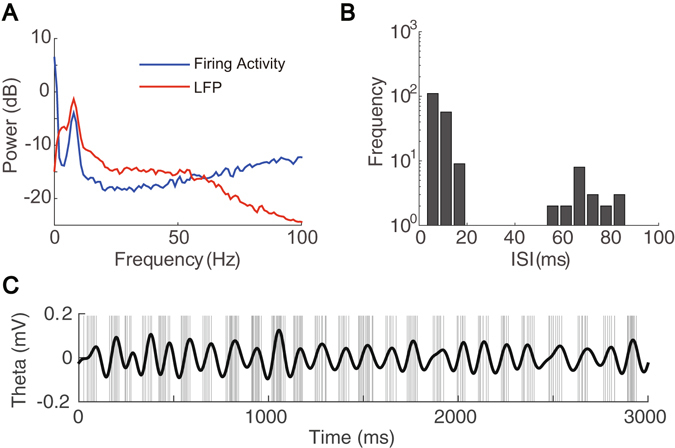
Properties of a theta-driving neuron. (**A**) Power spectra of a theta-driving neuron’s firing activity (blue) and the LFP signal (red). (**B**) The bimodal distribution of the ISI of the theta-driving neuron. (**C**) The time series of the theta-driving neuron’s firing activity and the corresponding theta wave. The time of each spike is indicated by a gray vertical line and the theta wave is plotted in black color. The theta-driving neuron tends to fire in the ascending phase of the theta wave.

To select the theta component, a power spectrum analysis is performed on both the firing activity of a theta-driving neuron and the simultaneously recorded LFP signal. There is a strong peak in both power spectra near 8 Hz (Fig. 1A), indicating a substantial theta rhythm in both signals. In addition, the distribution of the inter-spike intervals (ISI) of theta-driving neurons is bimodal, with one sharp peak around 5 ms and the other broad peak around 70 ms (Fig. 1B). The bimodal nature of the ISI distribution reflects the intraburst and interburst firing patterns of theta-driving neurons with a reliable theta-rhythmic burst of 5–15 spikes per theta cycle. By filtering the LFP signal with the theta band of 4–12 Hz to obtain a theta wave, we observe that the theta-driving neuron tends to fire in the ascending phase of the theta wave (Fig. 1C and Supplementary Fig. S1).

To confront the possibility that the interaction between theta-driving neurons and theta waves could be highly nonlinear, we apply the information theoretic approach to study the causal dependence between the firing activity of theta-driving neurons and the corresponding theta wave. As discussed in the *Introduction* and detailed in the *Materials and Methods*, we use the time-delayed mutual information to reveal the information flow between the firing activity of theta-driving neurons and the corresponding theta wave derived from the LFP signal.

**Figure 2:**
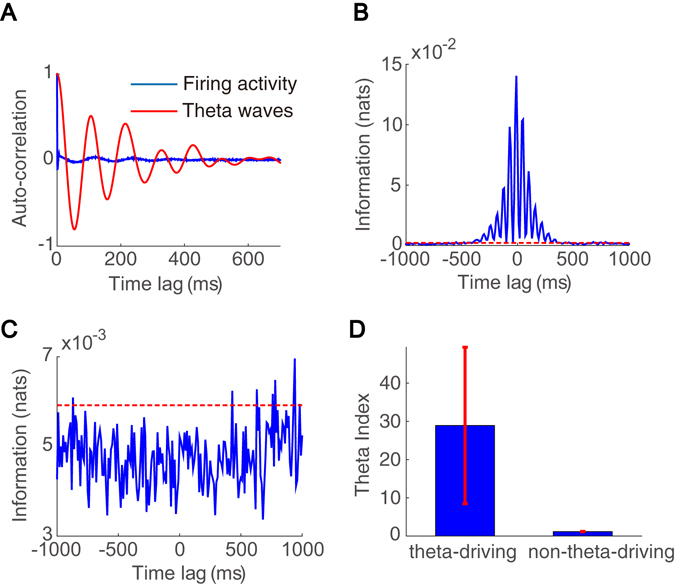
Interaction between theta-locked interneurons and theta waves in the AE state. (**A**) Auto-correlation of the theta-driving neuron’s firing activity (blue) and the corresponding theta wave (red). (**B**) Time-delayed mutual information between the firing activity of a theta-driving neuron and the corresponding theta wave. (**C**) Time-delayed mutual information between the firing activity of a theta-locked non-theta-driving neuron and the theta wave. In (**B**,**C**), we plot the mutual information as a function of time delay (blue) and its significance level (red). (**D**) Theta index for theta-driving neurons as well as non-theta-driving neurons. Blue bars and red bars indicate the mean and the range of theta index values for the six theta-driving neurons and four non-theta-driving neurons, respectively.

Typical data length in our analysis is 10–30 s, which is sufficiently long in comparison with the correlation time of the neuronal firing activity and the corresponding theta wave, which is less than 500 ms (Fig. 2A). We construct the probability distributions related to these two signals. Note that the distribution of the firing activity of theta-driving neurons is binary while the distribution of the amplitude of the theta wave is continuous. The amplitude of the theta wave is discretized into 30 bins. We then simply count the occurrence of each value from the time series of neuronal firing activity or the theta wave, respectively. The accuracy of the estimated probability distribution depends on the discretization level of the theta wave amplitude, the total number of data points, and the sampling rate of data in the experiment^28^. We comment that the conclusions below are insensitive to the variation of the number of discretization level from 30 to 100 as long as the data length used for probability construction is longer than 10 s.

Using the constructed probability distributions, we compute the time-delayed mutual information between the firing activity of theta-driving neurons and the corresponding theta wave during the active exploration (AE) state. As shown in Fig. 2B, the firing activity of a theta-driving neuron shares a substantial amount of mutual information with the theta wave. The value of time-delayed mutual information is substantially greater than the significance level (permutation test, $p < 0.05$). It exhibits an oscillatory pattern with frequency close to the theta band and decays to zero as the time-lag between the two signals increases. The oscillatory pattern results from the periodic behavior of the firing activity of the theta-driving neuron and the theta wave, and the decay of mutual information results from the fact that the neuronal signals are not perfectly periodic and have finite memory. Meanwhile, as shown in Fig. 2C, the firing activity of a non-theta-driving neuron shares little mutual information with the theta wave during the AE state. The value of mutual information is below the significance level (permutation test, $p < 0.05$) and it exhibits no discernable regular features. We note that the peak amplitude of the mutual information associated with the theta-driving neurons is one order of magnitude larger than those associated with non-theta-driving neurons, indicating there is a strong shared component of information between a theta-driving neuron and its corresponding theta wave in contrast to non-theta-driving neurons. Time-delayed mutual information functional profiles for other theta-driving neurons and non-theta-driving neurons can be found in Supplementary Figs S2 and S3, respectively. To quantify the significance of the mutual information measure, we define the theta index as the ratio of the peak amplitude in the mutual information as a function of time-lag to the significance level. All theta-driving neurons have a substantially greater theta index, ranging from 4.9 to 61.5 (n = 6), than non-theta-driving neurons, whose theta index ranges from 1.1 to 1.2 (n = 4) (Fig. 2D). Therefore, unlike non-theta-driving neurons, all the theta-driving neurons recorded in the experiments^28^ indeed interact strongly with the theta wave.

**Figure 3:**
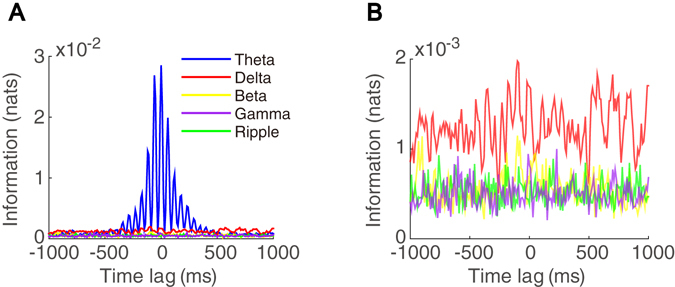
Interaction between a theta-driving neuron and waves over different frequency bands in the AE state. (**A**) Time-delayed mutual information between the waves of different frequency bands and the firing activity of a theta-driving neuron. The waves of the theta band (4–12 Hz) (blue), the delta band (1–4 Hz) (red), the beta band (12–30 Hz) (yellow), the gamma band (30–100 Hz) (purple), and the ripple band (100–250 Hz) (green) are obtained by filtering the original LFP signal. (**B**) Zoom-in of *A* but without the case of the theta band.

To investigate the interaction between a theta-driving neuron and waves of different frequency bands, we filter the LFP signal recorded during the AE state with the delta band (1–4 Hz), the beta band (12–30 Hz), the gamma band (30–100 Hz), and the ripple band (100–250 Hz) to obtain the delta wave, the beta wave, the gamma wave, and the ripple wave, respectively. We then compute the time-delayed mutual information between the firing activity of a theta-driving neuron and the waves of different frequency band respectively. From Fig. 3A, it is clear that the firing activity of a theta-driving neuron shares a substantially higher amount of information with the theta wave than with other waves. The peak amplitude of the mutual information with the theta wave is one order of magnitude larger than those corresponding to the delta, beta, gamma, and ripple waves (Fig. 3B). In this case, permutation test result shows that the mutual information associated with the beta, gamma, and ripple waves are significantly below their significance levels while the mutual information associated with the delta wave fluctuates around its significance level. Except for the case of the theta wave, all mutual information as a function of time-lag can be characterized as noise without any discernable regular features. Therefore, a theta-driving neuron interacts predominantly with the theta wave rather than the waves of other frequency bands during the AE state.

**Figure 4:**
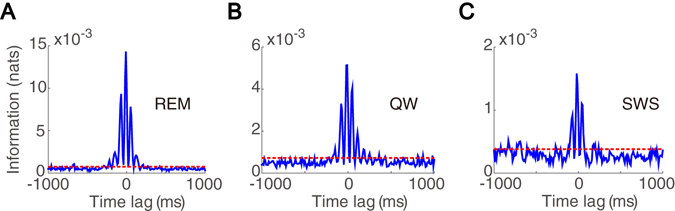
Interaction between a theta-driving neuron and the theta wave over different behavioral states. Time-delayed mutual information (blue) between the theta wave and the firing activity of a theta-driving neuron in the state of (**A**) REM, (**B**) QW, and (**C**) SWS. The red dashed line indicates the significance level.

To further study the interaction between a theta-driving neuron and the theta wave over different brain states, we separate a wake-sleep cycle into four characteristic behavioral states of active exploration (AE), quiet waking (QW), rapid-eye-movement (REM) sleep, and slow-wave sleep (SWS). As shown in Fig. 4, just as with the AE state, a theta-driving neuron also shares a substantial amount of mutual information with the theta wave in the REM, QW, and SWS states, far above the significance levels (permutation test, $p < 0.05$). Therefore, in all these behavioral states, a theta-driving neuron interacts strongly with the theta wave. We note that the shared component of information decreases from the AE and REM states to QW and SWS states (Fig. 4), which is consistent with the decrease of the power intensity of theta rhythm from the AE and REM states to QW and SWS states^28^.

To identify the direction of information flow between a theta-driving neuron and the theta wave, we examine the sign of the *peak time*-*lag*, which is the time-lag when the time-delayed mutual information reaches its peak amplitude (global maximum). We find that for five of the six theta-driving neurons the peak time-lag is −19.2 ± 3.0 ms (mean ± standard deviation) for the AE state. A negative peak time indicates that information is transferred from the firing activity of interneurons to the theta wave, thus *driving* the theta wave from the viewpoint of information transfer. In other words, the information embedded in theta waves in the hippocampus could originate from the firing activity of theta-driving neurons.

Out of the six theta-driving neurons examined, there is one theta-driving neuron that has a positive peak time about +1 ms. This particular neuron is thus likely influenced by the theta wave instead of the other way around. The influence could come indirectly from activities of other neurons that transfer information to both the theta wave and the activity of this neuron. If a neuron transfers information faster to the theta wave than to this theta-driving neuron, then the effective direction of information transfer could be from the theta wave to this neuron. Furthermore, we note that the mutual information as a function of time-lag for this neuron also exhibits a decaying oscillatory pattern (Supplementary Fig. S2F) with multiple local peaks at negative time lags. Similar to the case of the other five theta-driving neurons, these local peaks are likely to be induced by common memories shared in the neuronal activity and the theta wave. However, we cannot rule out the possibility that this theta-driving neuron also transfers information through its firing activity to the theta wave with a time delay signified by the local peaks. Theoretically, transfer entropy^32^ was developed to eliminate the effect of common history. However, in practice, the curse of dimensionality caused by the long-time memory together with the requirement of stationarity renders transfer entropy rather difficult to implement for our data as well as in many common experimental setups. We will further address the issue of transfer entropy in the *Discussion* section. Because we cannot ascertain whether this theta-driving neuron contributes to the generation of theta waves, we will exclude it in the following study.

### Classification of theta-driving neurons

In the previous section, we have shown that a theta-driving neuron interacts predominantly with the theta wave and the interaction is insensitive to behavioral states. The direction of information flow further indicates that five of the six theta-driving neurons *drive* theta waves. Although we have characterized the theta-driving neurons through interactions of their firing activity with theta waves, the question of what type of interneuron these theta-driving neurons belong to remains to be answered. In this section, we undertake the task of using realistic neuron simulation to classify theta-driving neurons as a particular type of interneurons, i.e., the identity of theta-driving neurons.

**Figure 5:**
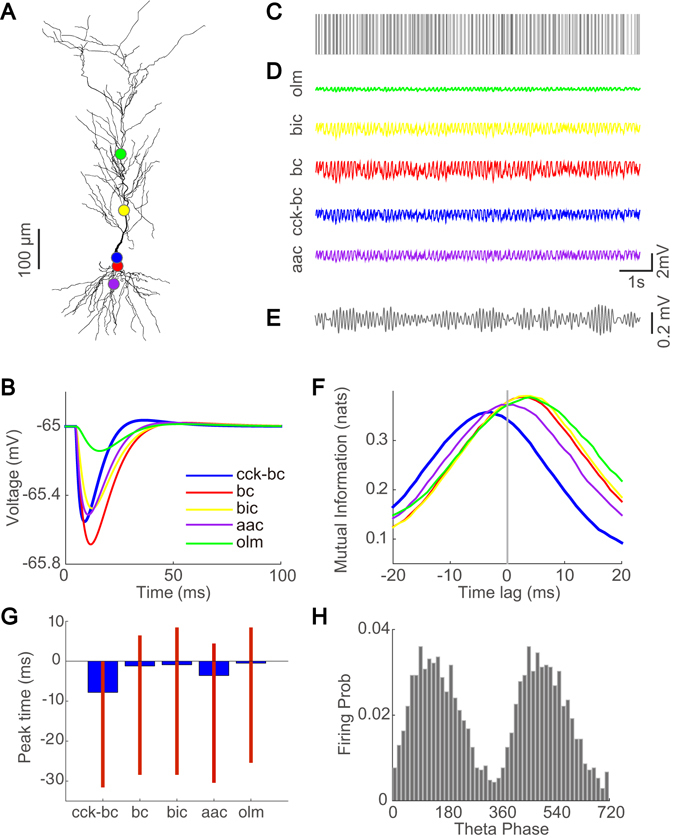
Classification of theta-driving neurons via realistic neuron simulations. (**A**) Morphology of the realistic CA1 pyramidal neuron model. Colored dots indicate the input locations of interneurons–AAC (purple), BC (red), CCK-BC (blue), BIC (yellow), and OLM (green). (**B**) Hyperpolarized somatic voltage trace of the pyramidal neuron in response to an individual input spike from an interneuron of each candidate type. (**C**) A spike train of a theta-driving neuron recorded in the experiment. (**D**) Computationally obtained hyperpolarized somatic voltage trace of the pyramidal neuron in response to the input from an interneuron of each candidate type. In our simulation, we assume that the spike train in *C* as the input to the pyramidal neuron is from an interneuron of one of the putative types with its input location and synaptic dynamics modeled with the corresponding parameters. (**E**) The theta wave obtained through filtering the LFP signal recorded in the experiment in the presence of the spike train of the theta-driving neuron shown in *C*. (**F**) Dependence of the time-lag of time-delayed mutual information between the experimentally recorded theta wave in *E* and the computationally obtained membrane potential induced by an interneuron of each type in *D*. (**G**) Peak time vs. interneuron type–peak time is the time-lag at which the time-delayed mutual information as a function of time-lag reaches its peak amplitude. Blue thick bars indicate the mean values of the peak time and red thin bars indicate the range of the peak time over 5 theta-driving neurons. (**H**) The firing probability of a theta-driving neuron as a function of the theta phase. In *A*, *B*, *D*, *F*, colors label different candidate interneuron types.

In early studies, interneurons of the BC, AAC, BIC, OLM, and CCK-BC types have been observed to fire phase-locked to theta waves^27^. Because theta-driving neurons also fire phase-locked to theta waves (Fig. 1C and Supplementary Fig. S1), it is natural to consider these five types of interneurons as candidates for our theta-driving neurons recorded in the experiment^28^. The interneurons of the five candidate types differ markedly in their postsynaptic dynamics and axonal target locations^33, 34^. The corresponding modeling parameters for each of these candidate interneuron types are introduced in *Materials and Methods*. The morphology of the realistic postsynaptic pyramidal neuron model adapted from refs 35–51. is shown in Fig. 5A. Using an individual spike as the input to the pyramidal neuron with the postsynaptic dynamics and location corresponding to a particular type of interneuron, our detailed neuronal simulation can generate the corresponding hyperpolarized postsynaptic membrane potential transients of the pyramidal neuron. As shown in Fig. 5B, the postsynaptic membrane potential transient induced by an interneuron of each candidate type displays distinct rise and decay time scales.

In the two-dipole model^17^, theta waves are generated by the interaction between two currents (dipoles) received by pyramidal neurons, i.e., perisomatic inhibition and distal dendritic excitation^52, 53^. Consistent with the two-dipole model, our previous results indicate that theta-driving neurons could be perisomatic interneurons, which shape theta oscillations. To further isolate theta-driving neurons to one of the five interneuron candidates, our key assumption is that *only* theta-driving neurons can induce a postsynaptic membrane potential change that further transfers information to theta waves. By using the experimentally recorded spike train of a theta-driving neuron (Fig. 5C) as the input to the realistic pyramidal neuron at the specific dendritic locations (Fig. 5A) in combination with distinct synaptic dynamics (Table 1) for each of the five interneuron candidates, we can simulate the corresponding hyperpolarized membrane potential trace in the pyramidal neuron (Fig. 5D) induced by each candidate interneuron. We emphasize that the input spike trains for the five cases are identical. The difference between the five thus obtained membrane potential traces arises from the different input locations and synaptic dynamics. In addition, we notice that the simulated membrane potential of the pyramidal neuron exhibits theta-rhythmic oscillations (Fig. 5D). This theta-rhythmic somatic hyperpolarization is induced by the spike train of a theta-driving neuron which is recorded in the presence of the theta wave. This simulation result is consistent with an early experimental observation^54^ that intracellular theta-rhythmic hyperpolarization in pyramidal neurons can occur in the presence of extracellular theta waves.

**Table 1:** Synaptic parameters of theta-driving neuron candidates.

Type	Rise Time (ms)	Decay Time (ms)	Axon Location	Distance* (*μ*m)
BC	1.8	13.7	soma	0
AAC	0.8	11.2	axon	25
BIC	2.0	16.1	dendrite	150
OLM	6.2	20.8	dendrite	300
CCK-BC	0.73	6.8	soma	0

*Here the distance is measured from the soma of the pyramidal neuron.

We next compute the time-delayed mutual information between the computationally obtained membrane potential of the pyramidal neuron (Fig. 5D) and the experimentally recorded theta wave (Fig. 5E) to obtain a mutual information curve for each of the five interneuron candidates. As shown in Fig. 5F, given the spike train of one specific theta-driving neuron (Fig. 5C), only the time-delayed mutual information between the theta wave and the membrane potential induced by CCK-BC yields a negative peak time-lag, indicating that only CCK-BC can consistently induce a potential information transfer from the activity of the pyramidal neuron to the theta wave.

We further compute the time-delayed mutual information between the simulated membrane potential of the pyramidal neuron and the theta wave using data recorded in multiple non-overlapping time segments. For each of such time segments, we obtain the peak time-lag of the time-delayed mutual information for each interneuron type. In Fig. 5G, we show that only the time-delayed mutual information for CCK-BC yields consistently a negative peak time-lag, indicating that only CCK-BC can induce a potential information transfer from the activity of the pyramidal neuron to the theta wave. This result suggests that the theta-driving neurons recorded in the experiment^28^ fall into the category of CCK-BC.

To further support our hypothesis that the theta-driving neurons are of the CCK-BC type, we perform the Hilbert transform of theta waves to obtain their phase in order to investigate the relation between the firing probability of a theta-driving neuron and the theta phase. As shown in Fig. 5H and Supplementary Fig. S1, the theta-driving neurons fire preferentially in the ascending phase of the theta wave. We note that the firing probability of these neurons as a function of theta phase is similar to that of the CCK neurons observed in an early experiment^27, 55^. This further corroborates our hypothesis. However, it should be pointed out that the early experiment^55^ was performed in anesthetized rats, different from ours, which was performed in freely behaving mice.

**Figure 6:**
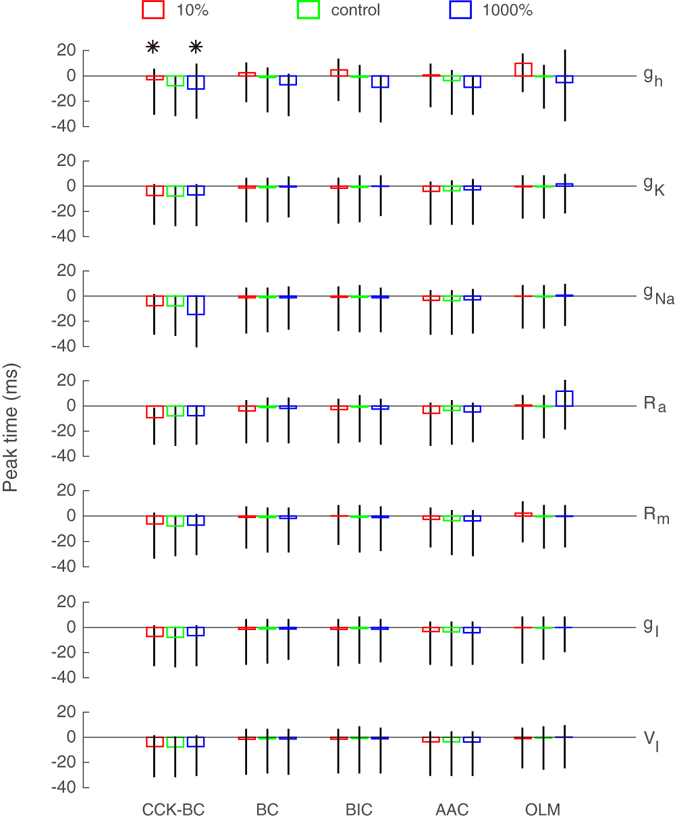
Sensitivity analysis of the parameters in the realistic pyramidal neuron model. Data of the five theta-driving neurons over 12 non-overlapping time segments are analyzed. Black bars indicate the range of the peak time and rectangle bars indicate the mean value of the peak time. Colors indicate the parameter range from 10% (red) to 1000% (blue) for all rows except the last one in which colors indicate the inhibitory reversal potential vary by increment of −10 mV (red) to +10 mV (blue) with respect to the control values (green) used in our realistic simulations. Asterisks are used to emphasize that the change of cation channel density *g*_*h*_ will lead to a substantial change of the positive peak time for CCK-BC.

From the results of our realistic neuron simulations, we hypothesize that the recorded theta-driving neurons belong to the CCK-BC type of interneurons. However, the validity of our conclusions relies on the model we use. The realistic pyramidal neuron model used above is merely a representative of pyramidal neurons. Therefore, sensitivity analysis is required to investigate how robust our conclusions are with respect to model parameters. The most important parameters in the model include the hyperpolarization-activated cation channel density *g*_*h*_, the potassium channel density ${g}_{K}$, the sodium channel density ${g}_{Na}$, the axial cytoplasmic resistance ${R}_{a}$, and the passive membrane resistance ${R}_{m}$. The values of these parameters in our model are derived from refs 39–51. We change each of these parameters from 10% to 1000% of the values we used above to examine the robustness of our conclusions. As shown in Fig. 6, varying each of the five parameters while fixing the remaining four parameters at the normal values as above results in a negative peak time of the time-delayed mutual information for CCK-BC. However, the peak time for other four interneuron candidates of BC, BIC, AAC, and OLM can vary from a positive to a negative value with large fluctuations, contradicting with our assumption that information flows consistently from postsynaptic membrane potentials to theta waves. In particular, the change of cation channel density *g*_*h*_ will lead to a substantial change of positive peak time even for CCK-BC (see asterisks in Fig. 6). It is consistent with early experiments^56^ which showed that the cation channel plays an important role in theta oscillations. In addition, the strength of the inhibitory conductance and the value of the inhibitory reversal potential for each interneuron type can vary over a large physiological range^57^. Therefore, we further vary the inhibitory conductance strength *g*_*I*_ from 10% to 1000% and increase the inhibitory reversal potential *V*_*I*_ from −10 mV to +10 mV with respect to the values we used above, and we find that the peak time of the time-delayed mutual information for CCK-BC stays almost unchanged and remains negative (Fig. 6). A pyramidal neuron *in vivo* may maintain its membrane potential at a high level after receiving numerous excitatory and inhibitory synaptic inputs. In such a state, the role of active dendritic conductances could differ from that in the state in our simulation. To account for this possibility, in our simulation, we also set the resting potential of the pyramidal neuron 10 mV greater than the value we used above (close to the firing threshold), and we find that our conclusion remains robust.

## Discussion

Hippocampal theta oscillations may be a substrate for cognitive computation. It is important to understand how information is processed through the interaction between interneurons and theta waves in the hippocampus. In this work, we have applied time-delayed mutual information to investigate the interaction between the theta wave and the firing activity of theta-driving neurons recorded in the hippocampal CA1 region of the freely behaving mouse. Our results have shown that the firing activity of theta-driving neurons shares a substantial amount of information with the theta wave, and the information flows from the activity of theta-driving neurons to the theta wave. The results suggest that theta-driving neurons transfer information to theta oscillations regardless of behavioral states. We further have performed realistic simulations of a CA1 pyramidal neuron model to address the question of which interneuron type these theta-driving neurons belong to. In particular, we have used time-delayed mutual information to analyze the information flow between the experimentally recorded theta wave and the computationally obtained membrane potential trace induced by each of five candidate interneurons–the BC, the AAC, the BIC, the OLM, and the CCK-BC. Based on the consistency of information flow from the activity of the pyramidal neuron to the theta wave, our analysis demonstrates that theta-driving neurons fit the characteristics of the CCK-BC.

Time-delayed mutual information analysis was initially developed and applied in the study of physical systems^58^. Later it has been introduced into neuroscience to measure the information transport^59^ and to infer the connectivity of neuronal networks^60, 61^. In contrast to many existing causal inference methods including Granger causality and cross-correlation, time-delayed mutual information analysis can capture the direction of information flow from one signal to the other for any nonlinear system, and it does not rely on the prior knowledge of the interaction form between the two signals. In addition, the quantity of mutual information is invariant under any nonlinear smooth and invertible transformation of signals. This enables mutual information to quantify the dependence of two signals independent of the measurement method of signals, even when the signals are obtained after certain attenuation and nonlinear transformation. In this work, by using time-delayed mutual information approach, we have characterized the interaction between theta-driving neurons and theta waves. In contrast to GC analysis^28^ that was unable to rule out the influence of LFP signals on the firing activity of non-theta-driving interneurons, here we have demonstrated that there is indeed no interaction between the non-theta-driving neuron and the theta wave. From our results so far, there is a sharp boundary between the feature of theta-driving neurons and non-theta-driving neurons. In the future study, we will explore the possibility of whether there exists intermediate types of theta related neurons by applying time-delayed mutual information in a larger data set.

In our work, we assumed that the signal of the neuronal activity or the LFP is a stationary process. The stationarity constrains the length of time series available for constructing the probability density function. The data needs to be relatively short to ensure a stationary brain state and sufficiently long to ensure a convergent construction of probability distributions. The data length is also required to be much longer than the characteristic correlation time of the signal (about 500 ms in our case above) in order to eliminate the memory effect of the signal. In our analysis above, we use data recorded during a single behavioral state to reasonably ensure stationarity with lengths typically ranging from 10 s to 30 s for the construction of probability distributions.

Because of the stationarity constraint, time-delayed mutual information is a better analysis tool in a practical setting than the other information theoretic approach known as transfer entropy^32^. Transfer entropy is a measure of information transfer from signal *X* to signal *Y* after excluding the influence of the Y’s own history. In principle, transfer entropy can eliminate the effect of common history of the two signals while time-delayed mutual information analysis cannot. In practice, however, transfer entropy requires much longer data lengths to construct the high-dimension probability density distributions. The dimension of probability distributions in transfer entropy is determined by the number of time lags between X and Y and that between Y and its own history, whereas the dimension of probability distribution in time-delayed mutual information analysis is only two. Therefore, compared with transfer entropy, time-delayed mutual information is a practicable approach under the requirements of stationarity and convergency of estimated probability distributions. Time-delayed mutual information is capable of identifying the existence of the interaction between two signals, regardless whether the interaction is linear or nonlinear.

The mechanism of the theta generation has been under active investigation over recent years. Experiments have shown that the medial septal area plays an important role in the theta generation^62^. Theoretically, a mechanism has been proposed for the generation of theta waves as a consequence of a feedback loop involving the medial septal area and hippocampus^63^. Therefore, theta waves may arise from the interaction between the pacemaker activity and resultant emergent dynamics in the network. Distinct from previous works, here we have studied the generation of theta waves from the viewpoint of information transfer. We emphasize that a theta-driving neuron transfers information to the theta wave but may not necessarily set theta waves as pacemakers.

We have shown that theta-driving neurons recorded in our experiments potentially are the CCK-BC neurons, because only CCK-BC out of the five types of candidate interneurons can consistently induce information flow from the activity of the pyramidal neuron to the theta wave. We further notice that those putative CCK-BC neurons exhibit the following features. By computing the time-delayed mutual information between the postsynaptic membrane potential induced by CCK-BC neurons and the theta wave, we observe that the value of the peak time-lag, which is always negative, can be further clustered into two groups–one is around −3 ms and the other is around −30 ms. For neurons with peak time-lag around −3 ms, they fire preferentially in the ascending phase near crests of the theta wave (Supplementary Fig. S1A–C) while for neurons with peak time-lag around −30 ms, they fire preferentially in the ascending phase near troughs of the theta wave (Supplementary Fig. S1D,E). The different delay time of information transfer from the membrane potential to the theta wave implies that there are possibly two distinct manifestations of the CCK-BC neurons in theta generation and information processing. It is crucial to carry out further studies of the properties of the CCK-BC as well as other interneurons to understand the detailed mechanisms underlying the generation and information processing of theta oscillations in the future.

## Methods

### Time-delayed mutual information

We use time-delayed mutual information to measure the functional dependence between the firing activity of interneurons and theta waves. In general, mutual information characterizes common information shared between two signals^64, 65^. Given signals *X* and *Y*, the mutual information between them is defined as

1 $$I(X,Y)=\sum _{x}\,\sum _{y}\,p(x,y)\,\mathrm{log}\,\frac{p(x,y)}{p(x)p(y)},$$

where $p(x,y)$ is the joint probability distribution of *X* and *Y*, $p(x)$ and $p(y)$ are their marginal probability distributions. In particular, *I*(*X*, *Y*) = 0 is equivalent to $p(x,y)=p(x)p(y)$, i.e., two signals *X* and *Y* are independent if they do not share information.

Mutual information is symmetric, i.e., *I*(*X*, *Y*) = *I*(*Y*, *X*). Therefore, it cannot be applied directly to detect the direction of information flow between two signals. To overcome this limitation, we introduce a time-lag parameter to capture information transfer between the two signals^58^. By calculating mutual information at various time lags, one can obtain a measure to infer the direction of information flow in general nonlinear systems. Time-delayed mutual information as a function of time-lag *τ* is defined as

2 $$I(X,Y,\tau )=\sum _{x}\,\sum _{y}\,p({x}_{t},{y}_{t+\tau })\,\mathrm{log}\,\frac{p({x}_{t},{y}_{t+\tau })}{p({x}_{t})\,p({y}_{t+\tau })},$$

where $p({x}_{t},{y}_{t+\tau })$ is the joint probability distribution of $X={x}_{t}$ and $Y={y}_{t+\tau }$. A large amplitude of the mutual information as a function of *τ* indicates the existence of functional interactions between two signals; the sign of the time-lag *τ* at the peak amplitude can be used to infer the information flow direction, which can be further interpreted as the direction of causality from the viewpoint of information transfer.

In contrast to cross-correlation or GC, which relies on linear characteristics of a system^30^, time-delayed mutual information can be applied to quantify nonlinear systems, for instance, neural systems. In addition, the evaluation of mutual information does not require any prior knowledge of the form of interactions and is invariant under any (nonlinear) smooth and invertible transformation of signals, i.e., independent of the method of the signal measurement. Therefore, it can be reliably applied to study causal interactions between two signals as long as the data length is sufficiently long for the construction of the probability distributions of the two signals.

### Data analysis

The experimental protocols were approved by the Experimental Animal Ethics Committee of East China Normal University (ethical review number AR201404009), and all experiments were performed in accordance with the university committee’s guidelines and regulations. Extracellular signals are recorded in the hippocampal CA1 area in freely behaving mice using a multi-electrode array. The experimental procedure follows what is published in ref. 28. The recording generally lasts for several hours. The signals recorded from electrodes are then filtered through preamplifiers to separate spike activity and LFP signal. Specifically, the spike activity (sampled at 40 kHz) and the LFP signal (sampled at 1 kHz) are filtered online at 400–7000 Hz and 0.7–300 Hz, respectively. Based on firing rates and the spike widths, spike sorting is carried out to classify each spike event to a specific neuron. We filter the LFP signal with the theta band (4–12 Hz) to obtain theta waves. In this work, we study the interaction between simultaneously recorded spike activity of interneurons and the theta waves.

At each moment of time, the neural activity is described by a binary variable indicating its firing or silent state. The amplitude of the theta wave is a continuous variable but discretized here into discrete values using 30 bins. For the data of spike activity and the theta wave, we estimate their joint probability distribution and the corresponding marginal probability distributions from their histograms. We have verified that our conclusions in the following are robust against the variation of the bin number from 30 to 100. To obtain reliable convergent probability distributions, the data lengths used for the construction of probability distributions are longer than 10 s so as to ensure there are more than 10^4^ sample points for constructing each probability distribution. Then, we calculate the time-delayed mutual information between the spike activity and the corresponding theta wave. Statistical significance is obtained with a permutation test by randomly shuffling the data of spike activity using the function *randperm*.*m* in MATLAB version 8.4. We shuffle the spike data 100 times and set the significance level as $p=0.05$.

### Realistic neuron simulation

We adapt the neuronal model in refs 35–51. for our realistic hippocampal CA1 pyramidal neuron simulation. The morphology of the reconstructed pyramidal neuron, which includes 200 compartments, is obtained from the Duke-Southampton Archive of Neuronal Morphology^38^. The passive cable properties and the densities of active conductances in the neuron model are based on published experimental data obtained from the hippocampal and cortical pyramidal neurons^39–51^.

We perform the simulation of the realistic pyramidal neuron to isolate the type of theta-driving neurons from a candidate set of five interneuron types, BC, BIC, AAC, OLM, and CCK-BC. The five candidate interneurons are distinct in their postsynaptic dynamics and their axonal target locations^33, 34^. The local postsynaptic conductances are modeled as the difference of two exponential functions^66^. Parameters corresponding to each candidate interneuron type are summarized in Table 1. In particular, BC and CCK-BC impinge on the soma of the pyramidal neuron with fast synaptic dynamics, BIC and OLM impinge on the apical dendrites with relatively slow synaptic dynamics, and AAC impinges on the axon with fast synaptic dynamics. The inhibitory reversal potential is set as −78 mV and the resting potential of the pyramidal neuron is set as −65 mV.

We use the NEURON software Version 7.3^67^ to simulate the model with time step of 0.1 ms. For each of the five interneuron candidates, by using the experimentally recorded spike train of a theta-driving neuron as the input to its corresponding axonal target location in the pyramidal neuron with its distinct synaptic dynamics (cf. Table 1), we numerically obtain the corresponding hyperpolarized membrane potential trace in the soma of the pyramidal neuron. We then analyze the time-delayed mutual information between the computationally obtained membrane potential and the experimentally recorded theta wave to infer to which one of the five interneuron types the theta-driving neuron belongs.

### Data Availability

The datasets generated during and/or analysed during the current study are available from the corresponding authors on reasonable request.

## Electronic supplementary material

Supplementary Information

## References

[1] O’Keefe J, Dostrovsky J (1971). The hippocampus as a spatial map. preliminary evidence from unit activity in the freely-moving rat. Brain research.

[2] McNaughton B, Barnes CA, O’keefe J (1983). The contributions of position, direction, and velocity to single unit activity in the hippocampus of freely-moving rats. Experimental Brain Research.

[3] Kahana MJ, Sekuler R, Caplan JB, Kirschen M, Madsen JR (1999). Human theta oscillations exhibit task dependence during virtual maze navigation. Nature.

[4] Grastyan E, Lissak K, Madarasz I, Donhoffer H (1959). Hippocampal electrical activity during the development of conditioned reflexes. Electroencephalography and clinical neurophysiology.

[5] Jouvet M (1969). Biogenic amines and the states of sleep. Science (New York, NY).

[6] Vanderwolf CH (1969). Hippocampal electrical activity and voluntary movement in the rat. Electroencephalography and clinical neurophysiology.

[7] Hasselmo ME, Bodelón C, Wyble BP (2002). A proposed function for hippocampal theta rhythm: separate phases of encoding and retrieval enhance reversal of prior learning. Neural computation.

[8] Benchenane K (2010). Coherent theta oscillations and reorganization of spike timing in the hippocampal-prefrontal network upon learning. Neuron.

[9] Lisman JE, Idiart MA (1995). Storage of 7 ± 2 short-term memories in oscillatory subcycles. Science.

[10] Buzsáki G, Moser EI (2013). Memory, navigation and theta rhythm in the hippocampal-entorhinal system. Nature neuroscience.

[11] Boyce R, Glasgow SD, Williams S, Adamantidis A (2016). Causal evidence for the role of rem sleep theta rhythm in contextual memory consolidation. Science.

[12] Hasselmo ME, Hay J, Ilyn M, Gorchetchnikov A (2002). Neuromodulation, theta rhythm and rat spatial navigation. Neural Networks.

[13] Buzsáki G (2005). Theta rhythm of navigation: link between path integration and landmark navigation, episodic and semantic memory. Hippocampus.

[14] Bland BH (1986). The physiology and pharmacology of hippocampal formation theta rhythms. Progress in neurobiology.

[15] Vinogradova O (1995). Expression, control, and probable functional significance of the neuronal theta-rhythm. Progress in neurobiology.

[16] Vertes R, Kocsis B (1997). Brainstem-diencephalo-septohippocampal systems controlling the theta rhythm of the hippocampus. Neuroscience.

[17] Buzsáki G (2002). Theta oscillations in the hippocampus. Neuron.

[18] Petsche H, Stumpf C, Gogolak G (1962). The significance of the rabbit’s septum as a relay station between the midbrain and the hippocampus i. the control of hippocampus arousal activity by the septum cells. Electroencephalography and clinical neurophysiology.

[19] Fox S (1989). Membrane potential and impedance changes in hippocampal pyramidal cells during theta rhythm. Experimental Brain Research.

[20] Kamondi A, Acsády L, Buzsáki G (1998). Dendritic spikes are enhanced by cooperative network activity in the intact hippocampus. The Journal of neuroscience.

[21] Wang X-J (2010). Neurophysiological and computational principles of cortical rhythms in cognition. Physiological reviews.

[22] Ranck JB (1973). Studies on single neurons in dorsal hippocampal formation and septum in unrestrained rats: Part i. behavioral correlates and firing repertoires. Experimental neurology.

[23] Fox SE, Ranck J (1975). Localization and anatomical identification of theta and complex spike cells in dorsal hippocampal formation of rats. Experimental neurology.

[24] Fox S, Ranck J (1981). Electrophysiological characteristics of hippocampal complex-spike cells and theta cells. Experimental Brain Research.

[25] Czurkó A, Huxter J, Li Y, Hangya B, Muller RU (2011). Theta phase classification of interneurons in the hippocampal formation of freely moving rats. The Journal of Neuroscience.

[26] Klausberger T (2003). Brain-state-and cell-type-specific firing of hippocampal interneurons *in vivo*. Nature.

[27] Klausberger T, Somogyi P (2008). Neuronal diversity and temporal dynamics: the unity of hippocampal circuit operations. Science.

[28] Zhang L (2012). Hippocampal theta-driving cells revealed by granger causality. Hippocampus.

[29] Granger, C. W. Investigating causal relations by econometric models and cross-spectral methods. *Econometrica*: *Journal of the Econometric Society* 424–438 (1969).

[30] Bressler SL, Seth AK (2011). Wiener–granger causality: a well established methodology. Neuroimage.

[31] Buzsáki G, Anastassiou CA, Koch C (2012). The origin of extracellular fields and currents-eeg, ecog, lfp and spikes. Nature reviews neuroscience.

[32] Schreiber T (2000). Measuring information transfer. Physical review letters.

[33] Maccaferri G (2000). Cell surface domain specific postsynaptic currents evoked by identified gabaergic neurones in rat hippocampus *in vitro*. The Journal of Physiology.

[34] Neu A, Földy C, Soltesz I (2007). Postsynaptic origin of cb1-dependent tonic inhibition of gaba release at cholecystokinin-positive basket cell to pyramidal cell synapses in the ca1 region of the rat hippocampus. The Journal of physiology.

[35] Hao J, Wang X, Dan Y, Poo M, Zhang X (2009). An arithmetic rule for spatial summation of excitatory and inhibitory inputs in pyramidal neurons. Proc Natl Acad Sci USA.

[36] Li S, Liu N, Zhang X-H, Zhou D, Cai D (2014). Bilinearity in spatiotemporal integration of synaptic inputs. PLoS Comput Biol.

[37] Li S, Zhou D, Cai D (2015). Analysis of the dendritic integration of excitatory and inhibitory inputs using cable models. Communications in Mathematical Sciences.

[38] Cannon R, Turner D, Pyapali G, Wheal H (1998). An on-line archive of reconstructed hippocampal neurons. Journal of neuroscience methods.

[39] Destexhe A, Mainen ZF, Sejnowski TJ (1994). An efficient method for computing synaptic conductances based on a kinetic model of receptor binding. Neural computation.

[40] Destexhe A, Mainen ZF, Sejnowski TJ (1994). Synthesis of models for excitable membranes, synaptic transmission and neuromodulation using a common kinetic formalism. Journal of computational neuroscience.

[41] Poirazi P, Brannon T, Mel BW (2003). Arithmetic of subthreshold synaptic summation in a model ca1 pyramidal cell. Neuron.

[42] Poirazi P, Brannon T, Mel BW (2003). Pyramidal neuron as two-layer neural network. Neuron.

[43] Stuart G, Spruston N (1998). Determinants of voltage attenuation in neocortical pyramidal neuron dendrites. The Journal of neuroscience.

[44] Magee JC, Johnston D (1995). Characterization of single voltage-gated na+ and ca2+ channels in apical dendrites of rat ca1 pyramidal neurons. The Journal of Physiology.

[45] Hoffman DA, Magee JC, Colbert CM, Johnston D (1997). K+ channel regulation of signal propagation in dendrites of hippocampal pyramidal neurons. Nature.

[46] Migliore M, Hoffman D, Magee J, Johnston D (1999). Role of an a-type k+ conductance in the back-propagation of action potentials in the dendrites of hippocampal pyramidal neurons. Journal of computational neuroscience.

[47] Magee JC (1998). Dendritic hyperpolarization-activated currents modify the integrative properties of hippocampal ca1 pyramidal neurons. The Journal of neuroscience.

[48] Magee JC, Cook EP (2000). Somatic epsp amplitude is independent of synapse location in hippocampal pyramidal neurons. Nature neuroscience.

[49] Andrásfalvy BK, Magee JC (2001). Distance-dependent increase in ampa receptor number in the dendrites of adult hippocampal ca1 pyramidal neurons. The Journal of Neuroscience.

[50] Smith MA, Ellis-Davies GC, Magee JC (2003). Mechanism of the distance-dependent scaling of schaffer collateral synapses in rat ca1 pyramidal neurons. The Journal of physiology.

[51] Nicholson DA (2006). Distance-dependent differences in synapse number and ampa receptor expression in hippocampal ca1 pyramidal neurons. Neuron.

[52] Kocsis B, Bragin A, Buzsáki G (1999). Interdependence of multiple theta generators in the hippocampus: a partial coherence analysis. The Journal of neuroscience.

[53] Montgomery SM, Betancur MI, Buzsáki G (2009). Behavior-dependent coordination of multiple theta dipoles in the hippocampus. The Journal of Neuroscience.

[54] Kamondi A, Acsády L, Wang X-J, Buzsáki G (1998). Theta oscillations in somata and dendrites of hippocampal pyramidal cells *in vivo*: Activity-dependent phase-precession of action potentials. Hippocampus.

[55] Klausberger T (2005). Complementary roles of cholecystokinin-and parvalbumin-expressing gabaergic neurons in hippocampal network oscillations. The Journal of neuroscience.

[56] Stark E (2013). Inhibition-induced theta resonance in cortical circuits. Neuron.

[57] Woodruff A, Xu Q, Anderson SA, Yuste R (2009). Depolarizing effect of neocortical chandelier neurons. Frontiers in neural circuits.

[58] Vastano JA, Swinney HL (1988). Information transport in spatiotemporal systems. Physical Review Letters.

[59] Destexhe A (1994). Oscillations, complex spatiotemporal behavior, and information transport in networks of excitatory and inhibitory neurons. Physical Review E.

[60] Wilmer A, de Lussanet M, Lappe M (2012). Time-delayed mutual information of the phase as a measure of functional connectivity. PloS one.

[61] Endo W, Santos FP, Simpson D, Maciel CD, Newland PL (2015). Delayed mutual information infers patterns of synaptic connectivity in a proprioceptive neural network. Journal of computational neuroscience.

[62] Stewart M, Fox SE (1990). Do septal neurons pace the hippocampal theta rhythm?. Trends in neurosciences.

[63] Wang X-J (2002). Pacemaker neurons for the theta rhythm and their synchronization in the septohippocampal reciprocal loop. Journal of Neurophysiology.

[64] Shannon CE (2001). A mathematical theory of communication. ACM SIGMOBILE Mobile Computing and Communications Review.

[65] Cover, T. M. & Thomas, J. A. *Elements of information theory* (John Wiley & Sons, 2012).

[66] Dayan, P. & Abbott, L. F. *Theoretical neuroscience* (The MIT Press, 2001).

[67] Carnevale, N. & Hines, M. *The NEURON book* (Cambridge: Cambridge Univ. Press, 2006).

