# Supplementary material for: The characterization of hippocampal theta-driving neurons — a time-delayed mutual information approach
Source: Sci Rep. 2017 Jul 17;7:5637. doi: 10.1038/s41598-017-05527-2 (PMC5514076; doi:10.1038/s41598-017-05527-2)
Supplement: Supplementary file 1 — Supplementary Information [file 41598_2017_5527_MOESM1_ESM.pdf]

Supplementary Information: The characterization of hippocampal theta-driving neurons — a time-delayed mutual information approach

Songting Li[1,+], Jiamin Xu[2,+], Guifen Chen[2], Longnian Lin[2,*], Douglas Zhou[3,†], and David Cai[1,3,4,§]

[1]Courant Institute of Mathematical Sciences and Center for Neural Science, New York University, New York, NY, United States of America
[2]Shanghai Key Laboratory of Brain Functional Genomics (Ministry of Education), School of Life Science and the Collaborative Innovation Center for Brain Science, Institute of Brain Functional Genomics, East China Normal University, Shanghai, China
[3]School of Mathematical Sciences, MOE-LSC, and Institute of Natural Sciences, Shanghai Jiao Tong University, Shanghai, China
[4]NYUAD Institute, New York University Abu Dhabi, Abu Dhabi, United Arab Emirates
[*]lnlin@brain.ecnu.edu.cn
[†]zdz@sjtu.edu.cn
[§]cai@cims.nyu.edu
[+]these authors contributed equally to this work

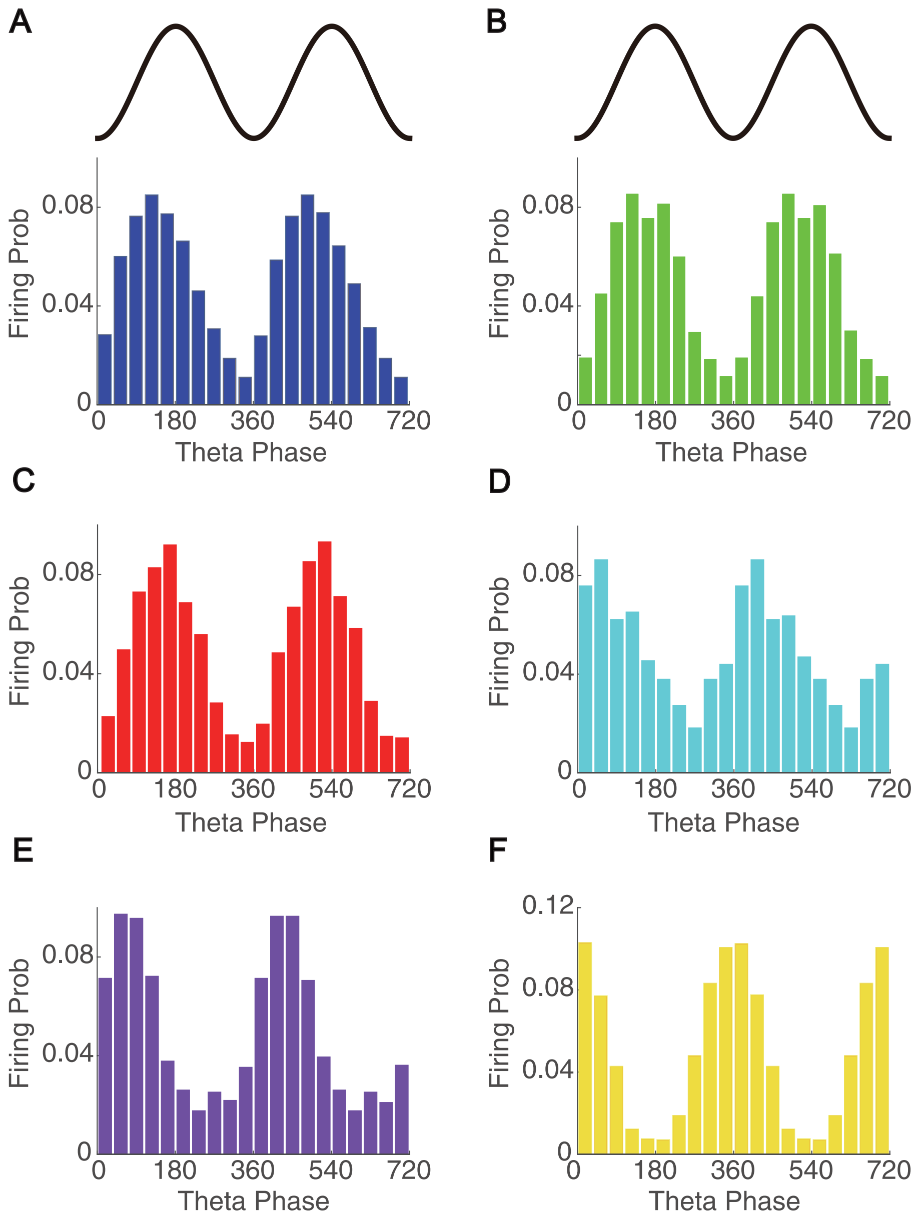

Figure S1. The firing probability as a function of the theta phase for the six theta-driving neurons. Top is the reference theta cycles. The firing probability is labeled with a distinct color for each neuron. A specific neuron is coded by the same color as in Fig. S2.

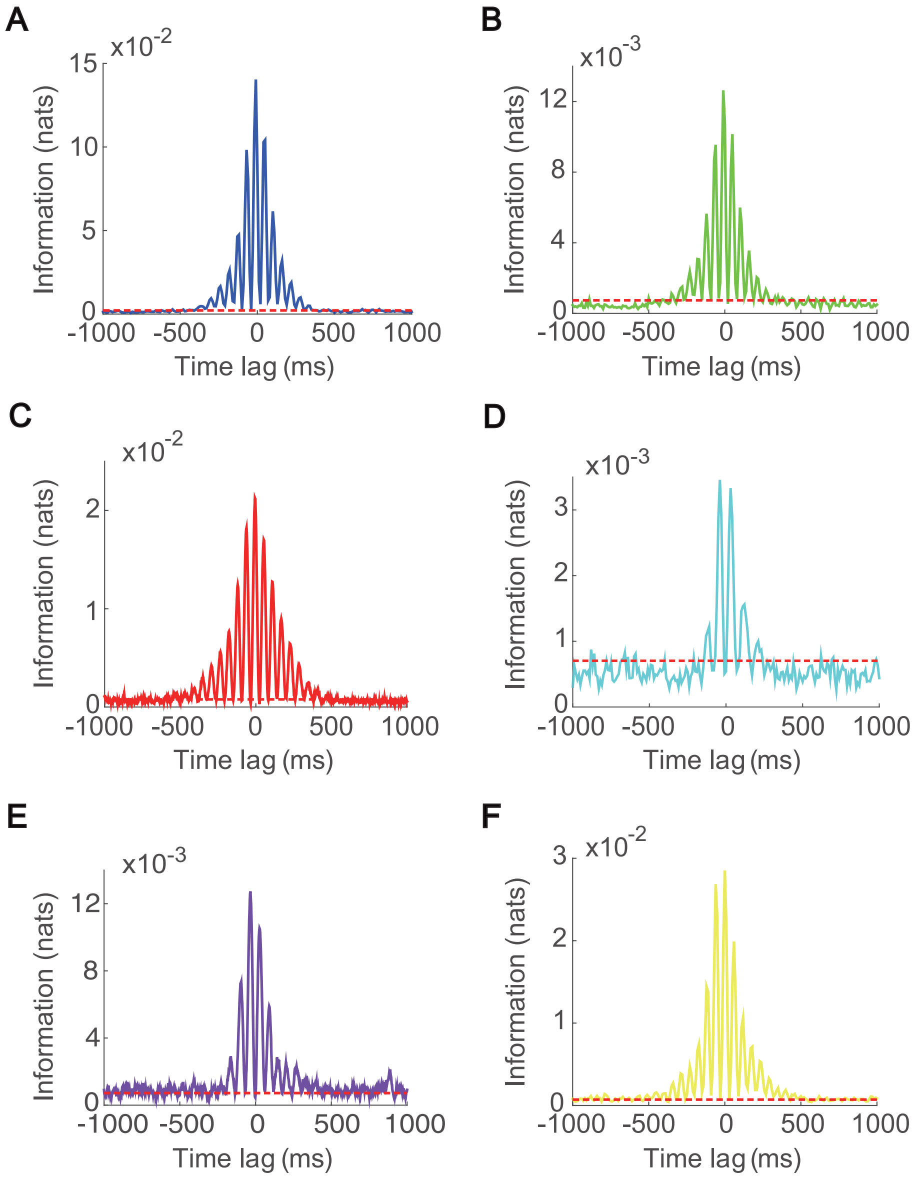

Figure S2. Interaction between a theta-driving neuron and the theta wave. Shown here is the time-delayed mutual information between the firing activity and the corresponding theta wave as a function of time-lag for the six theta-driving neurons. The time-delayed mutual information is color-coded: A color codes a specific theta-driving neuron and the significance level is indicated by the red dashed line. A specific neuron is coded by the same color as in Fig. S1.

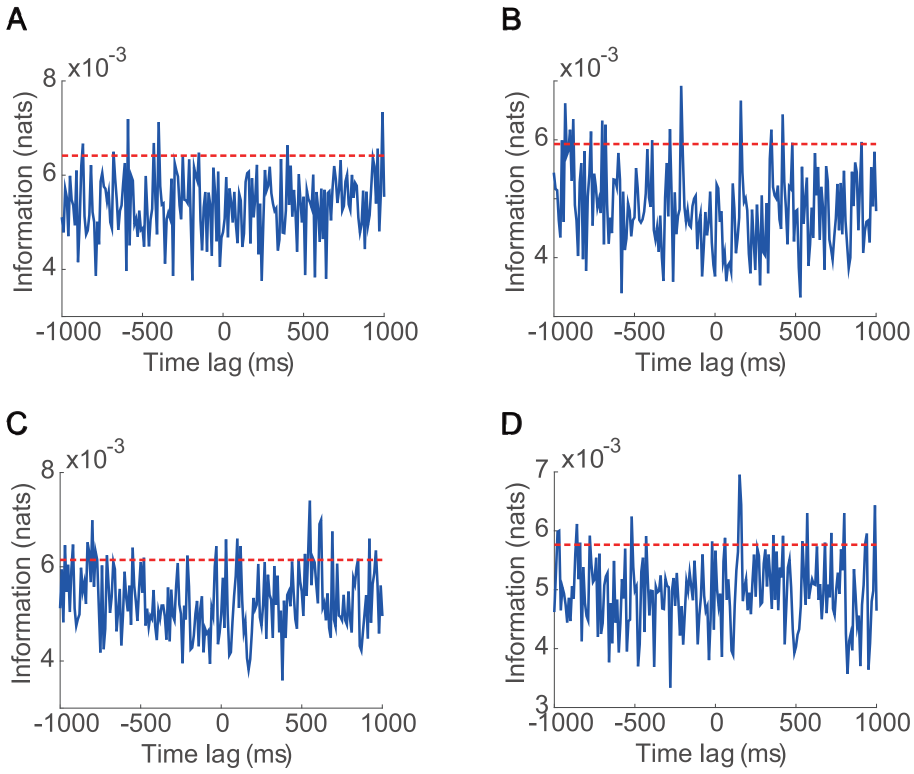

Figure S3. Interactions between a non-theta-driving neuron and the theta wave. (A-D) Time-delayed mutual information between the firing activity of a non-theta-driving neuron and its corresponding theta wave as a function of time-lag. Four cases of non-theta-driving neurons are shown. The time-delayed mutual information is in blue color and the significance level is in red color.
